# Supplementary material for: Paternal Exposure to Bisphenol-A Transgenerationally Impairs Testis Morphology, Germ Cell Associations, and Stemness Properties of Mouse Spermatogonial Stem Cells
Source: Int J Mol Sci. 2020 Jul 29;21(15):5408. doi: 10.3390/ijms21155408 (PMC7432732; doi:10.3390/ijms21155408)
Supplement: Supplementary file 1 [file ijms-21-05408-s001.pdf]

International Journal of *Molecular Sciences*

MDPI

Article

Paternal Exposure to Bisphenol-A Transgenerationally impairs Testis Morphology, Germ Cell Associations, and Stemness Properties of Mouse Spermatogonial Stem Cells

Polash Chandra Karmakar [1,†], Jin Seop Ahn [1,†], Yong-Hee Kim [1], Sang-Eun Jung [1], Bang-Jin Kim [2], Hee-Seok Lee [3], Sun-Uk Kim [4], Md Saidur Rahman [1], Myung-Geol Pang [1], and Buom-Yong Ryu [1,*]

[1] Department of Animal Science and Technology and BET Research Institute, Chung-Ang University, Anseong, 17546, Republic of Korea; polashmicro@gmail.com (P.C.K), ahnjs@cau.ac.kr (J.S.A), yhkcau@naver.com (Y.-H.K), tkddms2428@naver.com (S.-E.J), shohagvet@gmail.com (M.S.R), mgpang@cau.ac.kr (M.-G.P)

[2] Department of Cancer Biology, Perelman School of Medicine, University of Pennsylvania, Philadelphia, PA, 19104, USA; bakim430@gmail.com (B.-J.K)

[3] Department of Food Science & Technology, Chung-Ang University, Anseong, 17546, Republic of Korea; hslee0515@cau.ac.kr (H.-S.L)

[4] National Primate Research Center and Futuristic Animal Resource and Research Center, Korea Research Institute of Bioscience and Biotechnology (KRIBB), Ochang, 28116, Republic of Korea; sunuk@kribb.re.kr (S.-U.K)

*Correspondence: byryu@cau.ac.kr (B.-Y.R); Tel: +82-31-670-4687, Fax: +82-31-670-0062

† These authors contributed equally to this work.

Supplementary Tables

Table S1. Proportion of germ cells in the testis of F0 to F4 generations

Generation	Treatment group	Total Spermatids (1C)	Spermatogonia[†] (2C)	Primary spermatocytes[††] (4C)
F0	Control	44.50 ± 1.23	16.85 ± 0.78	13.56 ± 0.87
	NOAEL	43.33 ± 1.66	16.24 ± 0.94	11.48 ± 0.89
	LOAEL	53.24 ± 1.50**	13.21 ± 0.52*	8.82 ± 0.91**
	EE	50.63 ± 1.68*	13.71 ± 0.84*	10.05 ± 0.85*
F1	Control	41.77 ± 0.78	16.34 ± 0.74	13.36 ± 0.76
	NOAEL	42.30 ± 0.88	13.33 ± 0.87*	11.93 ± 0.82
	LOAEL	48.92 ± 1.65*	12.91 ± 0.61*	9.93 ± 0.5
	EE	49.03 ± 1.58*	13.01 ± 0.67*	9.9 ± 0.76
F2	Control	43.10 ± 1.40	17.25 ± 0.60	13.4 ± 0.81
	NOAEL	44.33 ± 1.47	15.54 ± 0.79	14.02 ± 1.00
	LOAEL	51.24 ± 2.28*	15.01 ± 0.62	11.62 ± 0.72
	EE	49.63 ± 2.08	15.01 ± 0.76	9.98 ± 0.81*
F3	Control	41.94 ± 0.13	13.11 ± 0.23	12.07 ± 0.17
	NOAEL	41.66 ± 0.14	14.02 ± 0.37	11.22 ± 0.32
	LOAEL	42.18 ± 0.51	12.27 ± 0.21	10.55 ± 0.69
	EE	44.11 ± 0.24	11.01 ± 0.32	9.01 ± 0.29
F4	Control	41.04 ± 0.13	14.11 ± 0.29	14.07 ± 0.17
	NOAEL	41.66 ± 0.14	14.02 ± 0.36	12.25 ± 0.3
	LOAEL	42.18 ± 0.11	13.17 ± 0.41	12.55 ± 0.69
	EE	41.18 ± 0.34	12.84 ± 0.35	11.01 ± 0.29

[†] somatic cells like spermatogonia and secondary spermatocytes

[††] G2/M and primary spermatocytes

*$p < 0.05$ and ** $p < 0.01$

Supplementary Figure

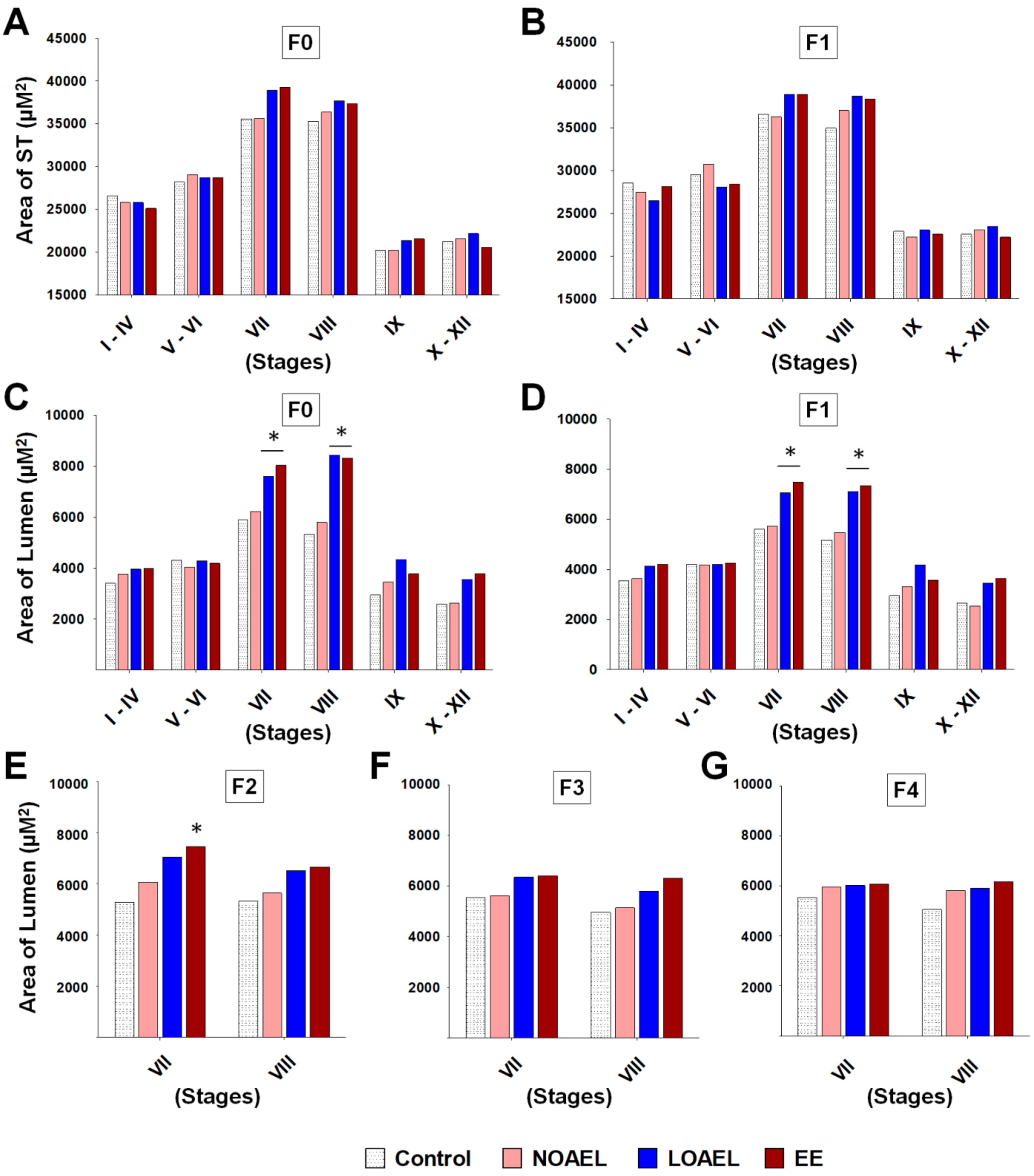

Figure S1. Area of seminiferous tubules and lumen. As the area of seminiferous tubules (STs) and lumen varies according to the stages of seminiferous epithelium (SE), the graphs indicate the STs and lumen area at different SE stages. The area of seminiferous tubules (STs) in (A) exposed group (F0) and (B) F1 generation was measured in μm^2. The area of lumen (μm^2) is shown in (C) F0 and (D) F1 generation. Around 15 mice/group were used for this study. The area of lumen (μm^2) is displayed for (E) F2, (F) F3, and (G) F4 generation (n = 9 mice/group). Data were analyzed by one-way analysis of variance (ANOVA) and asterisk (*) indicates a statistically significant difference between the exposure groups and the control group in the same stage of SE (*$p < 0.05$).
